# Antimicrobial Effectiveness of Calcium Silicate Sealers against a Nutrient-Stressed Multispecies Biofilm

**DOI:** 10.3390/jcm9092722

**Published:** 2020-08-24

**Authors:** Rahul Bose, Konstantinos Ioannidis, Federico Foschi, Abdulaziz Bakhsh, Robert D. Kelly, Sanjukta Deb, Francesco Mannocci, Sadia Ambreen Niazi

**Affiliations:** 1 Centre for Host Microbiome Interactions, King’s College London Dental Institute, Floor 17, Tower Wing, Guy’s Dental Hospital, London Bridge, London SE1 9RT, UK; rahul.bose@kcl.ac.uk (R.B.); konstantinos.ioannidis@kcl.ac.uk (K.I.); federico.foschi@kcl.ac.uk (F.F.); abdulaziz.bakhsh@kcl.ac.uk (A.B.); kellyr6@tcd.ie (R.D.K.); sanjukta.deb@kcl.ac.uk (S.D.); francesco.mannocci@kcl.ac.uk (F.M.); 2 Department of Therapeutic Dentistry I. M., Sechenov First Moscow State Medical University, 119146 Moscow, Russia; 3 Department of Restorative Dentistry, Faculty of Dentistry, Umm Al-Qura University, Makkah 24381, Saudi Arabia

**Keywords:** antimicrobial properties, bioceramic sealers, biofilms, calcium silicate sealers, confocal laser scanning microscopy, dental materials, endodontic sealers

## Abstract

Purpose: This study compared the antimicrobial efficacy of calcium silicate sealers (BioRoot RCS and Total Fill BC) and conventional sealers (AH Plus and Tubli-seal) against planktonic bacteria and a nutrient-stressed multispecies biofilm. Methods: Antimicrobial properties of freshly mixed sealers were investigated using the direct contact test (DCT) and a nutrient-stressed multispecies biofilm comprised of five endodontic strains. Antimicrobial activity was determined using quantitative viable counts and confocal laser scanning microscopy (CLSM) analysis with live/dead staining. The pH of the sealers was analysed over a period of 28 days in Hanks Balanced Salt Solution (HBSS). Analysis of variance (ANOVA) with Tukey tests and the Kruskal–Wallis test were used for data analysis with a significance of 5%. Results: All endodontic sealers exhibited significant antimicrobial activity against planktonic bacteria (*p* < 0.05). BioRoot RCS caused a significant reduction in viable counts of the biofilms compared to AH Plus and the control (*p* < 0.05), while no significant difference could be observed compared to TotalFill BC and Tubli-seal (*p* > 0.05). CLSM analysis showed that BioRoot RCS and TotalFill BC exhibited significant biofilm inhibition compared to Tubli-seal, AH Plus and the control (*p* < 0.05). BioRoot RCS presented with the highest microbial killing, followed by TotalFill BC and Tubli-seal. Alkalizing activity was seen from the onset by BioRoot RCS, TotalFill BC and AH Plus. After 28 days, BioRoot RCS demonstrated the highest pH in HBSS (pH > 12). Conclusions: Calcium silicate sealers exhibited effective antimicrobial properties. This was demonstrated by superior biofilm inhibition capacity and microbial killing, with strong alkalizing activity compared to epoxy-based and zinc oxide-eugenol-based sealers.

## 1. Introduction

The primary aetiology of endodontic disease is the microbial infection of the root canal system [1,2]. Multispecies bacterial colonies exist within the infected root canal in the form of biofilms [3], making them resistant to antimicrobial agents [4]. The complete removal of the root canal biofilms is challenging due to the complex anatomy of the root canal system, resulting in the inhibition of adequate irrigant penetration and contact with the biofilm [5]. Hence, residual microbes remaining at the obturation stage can have a significant effect on the outcome of endodontic treatment, leading to persisting infections [6]. Similarly, microbial coronal leakage into obturated root canals may cause reinfection and endodontic treatment failure [7,8,9]. Root canal sealers are required to seal the interface between the obturating core and root canal dentine, as well as the structural voids within the obturation material. Furthermore, sealers exhibiting antimicrobial action may aid in the reduction of residual and incoming microbes, thereby improving the endodontic treatment outcome [10]. Calcium silicate sealers have been commercially available for over a decade and are mainly based on tricalcium silicate, dicalcium silicate and tricalcium phosphate. They have been shown to have superior bioactive and physiochemical in comparison to zinc oxide-eugenol and epoxy resin-based sealers [11,12,13].The antimicrobial efficacy of hydraulic calcium silicate sealers has been investigated using in vitro tests, including the direct contact test (DCT) [11,14] and mono-species biofilm models, reporting promising antimicrobial activity of calcium silicate sealers [15,16,17,18]. However, limited information is available in the literature regarding the antimicrobial effectiveness of calcium silicate sealers on multispecies biofilm, which is of clinical relevance to the actual endodontic microbiota [3,19]. An in vitro, nutrient-stressed multispecies endodontic biofilm model has been developed and used in previous studies [20,21]. This biofilm model simulates the in vivo environment of endodontic infections by being mature and polymicrobial and including nutritionally stressed microorganisms relevant to the endodontic microbiome.

The aim of this study was to investigate in vitro the antimicrobial activity of calcium silicate and conventional endodontic sealers against planktonic microbes and the aforementioned nutrient-stressed multispecies biofilm model. The secondary aim was to analyse the pH value of the sealers over a period of 28 days.

## 2. Material and Methods

For the purposes of this study, two commercially available calcium silicate sealers, Total Fill BC (FKG swiss Endo, La Chaux-de-Fonds, Switzerland) (Group 1) and BioRoot RCS (Septodont, Saint Maur-des-Fossess, France) (Group 2), were used along with AH Plus (Dentsply DeTrey, Kontanz, Germany) (Group 3), an epoxy-based sealer, and Tubli-seal (Kerr Italia, Salerno, Italy) (Group4), a zinc oxide-eugenol based sealer. The chemical composition of the sealers is shown in Table 1. Total Fill BC, AH Plus and Tubli-seal were available as premixed sealers. In the case of BioRoot RCS, the powder and liquid were prepared according to the manufacturers’ instructions using a sterile spatula and sterile mixing pad inside a class II type B2 biological safety cabinet (Thermo-Fischer, Scientific, Loughborough, UK).

### 2.1. Direct Contact Test

The antimicrobial activity of each sealer was evaluated against planktonic suspensions of five microbial strains. The selected endodontic microbes included Propionibacterium acnes (*P. acnes*), Actinomyces radicidentis (*A. radicidentis*), Staphylococcus epidermidis (*S. epidermidis*), Streptococcus mitis/oralis (*S. mitis/oralis*) recovered from root canals of teeth with refractory endodontic infections and Enterococcus faecalis (*E. faecalis*) strain OMGS 3202 [22,23]. All bacteria were cultured anaerobically at 37 °C for seven days on Fastidious Anaerobic Agar (FAA) supplemented with 5% Horse Blood (FAA, Thermo Scientific™, Loughborough, UK). One loopful of the bacterial culture was inoculated into 1 mL of brain heart infusion (BHI) broth (Brain-Heart Infusion Broth, Lab M, Bury, UK) and the absorbance was adjusted with BHI to 0.25 at 550-nm optical density to obtain bacterial concentrations of 3 × 10^8^ mL using a Labsystem iEMS Reader (MF, Basingstoke, UK). In each experimental group (1–4), 5 subgroups were assigned, based on each microbial species. Of each freshly prepared sealer (*n* = 60, for each sealer group assigned to 5 bacteria used in this study, with the experiment performed in triplicates), 200 μL was injected in the base of individual well of sterile 24-well flat bottom plate (StarLab, Milton Keynes, UK). Separate 24-well flat bottom plates (Star Lab, Milton Keynes, UK) were used for each sealer group. Immediately after sealer insertion into the wells, 500 μL of bacterial suspension was added on the top of each sealer. To ensure the appropriate microbial growth, in a positive control group, the bacterial suspensions (*n* = 5, for each bacterial species) were injected in the wells, with no sealer addition. A negative control group (*n* = 5) was also assigned to ensure the absence of cross-contamination in culture medium. This included the injection of previously sterilised BHI into empty wells. The 24-well plates were incubated anaerobically at 37 °C for seven days in an anaerobic workstation (MACS-MG-1000, Don Whitley Scientific Ltd., Bingley, UK).The quantitative viable counts were determined by performing serial dilution, and 100-μL aliquots were plated onto triplicated FAA plates supplemented with 5% Horse Blood (FAA, Thermo Scientific™, Loughborough, UK). FAA plates were then incubated anaerobically at 37 °C for seven days. After this period, the colony forming units (CFU) and their log10 (log10CFU) were counted. The analysis was performed at different time intervals including T1 (1 h), T2 (24 h) and T3 (168 h) after direct contact between the microbial suspension and sealer and T3 for the control groups.

### 2.2. Multispecies Biofilm Inhibition Test

#### 2.2.1. Specimen Preparation

For the 4 sealer groups, 12 discs (*n* = 3 per group; experiment performed in triplicates) were prepared using a Teflon mould (10-mm diameter, 1-mm thickness). The sealers were prepared as previously mentioned and inserted into the moulds. The specimens were stored at 37 °C and 95 ± 5% relative humidity (Memmert, Thermo Scientific™, Loughborough, UK) as per the manufacturers’ stated setting times for each sealer (Table 2). Following setting, the specimens were removed from the mould using sterile tweezers and exposed to ultraviolet (UV) radiation (AirClean 600 Series PCR Workstations, AirClean Systems, Creedmoor, NC, USA) for 60 min. The discs were repositioned every 20 min to ensure full surface exposure. Sterilised hydroxyapatite (HA) discs (3D Biotek, Bridgewater, NJ, USA) were used as a positive control group (*n* = 3) and a negative control group (*n* = 3). All discs (*n* = 18) were placed in 3 mL of modified fluid universal medium (mFUM) [24] contained in a 24-well tray (Star Lab, Milton Keynes, UK) and pre-reduced in an anaerobic workstation (MACS-MG-1000, Don Whitley Scientific Ltd., Bingley, UK).

#### 2.2.2. Multispecies Biofilm Growth

A nutrient-stressed multispecies biofilm comprising of five selected microbes was grown on the surfaces of the sealer discs using the protocol developed by Niazi et al. [21] for the biofilm inhibition test. The endodontic strains, recovered from root canals of teeth with refractory endodontic infections, included *Propionibacterium acnes*, *Actinomyces radicidentis*, *Staphylococcus epidermidis*, *Streptococcus mitis/oralis,* and *Enterococcus faecalis* strain OMGS 3202 [22,23]. The individual microbial strains were cultured anaerobically at 37 °C for seven days on Fastidious Anaerobe Agar (FAA) supplemented with 5% defibrinated horse blood (FAA, Thermo Scientific™, Loughborough, UK). The 5 individual microbial starter culture was then transferred, in an anaerobic workstation (MACS-MG-1000, Don Whitley Scientific Ltd., Bingley, UK), into 3 mL of mFUM [24] and incubated at 37 °C for 3 h. The absorbance was adjusted to 0.5 at 540 nm to obtain 10^7^ cells/mL^−1^ using Labsystems iEMS Reader (MF, Basingstoke, Bingley, UK). All sealer discs (*n* = 12) were seeded with 400 μL of each of the five individual starter cultures. The biofilm was grown with regular medium change every 24 h for the first seven days in an anaerobic workstation. The biofilm was starved for another seven days without medium change. Concurrently, the same methodology was utilised in the control group to establish biofilms. In the positive control group (*n* = 3), biofilm growth was established on HA discs (3D Biotek, Bridgewater, NJ, USA), without insertion of endodontic sealer. In the negative control group (*n* = 3), the hydroxyapatite discs (3D Biotek, Bridgewater, NJ, USA) were immersed in sterilised mFUM to confirm the absence of contamination.

#### 2.2.3. Determination of Quantitative Viable Counts of the Biofilms after 14 Days

After 14 days, all discs were removed from the anaerobic workstation and their suspension. Each disc was transferred into 1 mL of BHI (brain-heart infusion broth, Lab M, Bury, UK) using a sterile tweezer and vortexed with sterilised 0.1-mm glass beads (Scientific Industries Inc., Bohemia, NY, USA) for 2 min, to allow for dispersion of the biofilm from the surface of the disc. To enumerate the microbial counts, serial dilution in BHI was performed and 100-μL aliquots were plated onto triplicate FAA plates and anaerobically incubated for seven days. After the incubation period, the number of colonies and their log_10_ (log_10_ CFU) were counted.

#### 2.2.4. Confocal Laser Scanning Microscopy (CLSM) Analysis of the Multispecies Biofilm

For this analysis, the nutrient-stressed multispecies biofilm was grown on the surface of the sealer discs as mentioned in Section 2.2.2. Three specimens from each sealer group was gently washed with phosphate-buffered saline (PBS) to remove nonadherent cells, stained with Live/Dead BacLight bacterial viability kit (Thermofisher Scientific, Loughborough, UK) and rewashed before analysis under an inverted Leica TCS SP2 CLSM (Leica Microsystems, Milton Keynes, UK). The disc specimens were transferred into 35-mm cell imaging coverglass-bottom dishes (SPL LifeSciences, Gyeonggi-do, Korea) with the biofilm embedded surfaces toward the objective. A × 63 magnification oil immersion objective with a numerical aperture of 1.40 and a confocal pinhole to Airy 1 unit was used to observe the fluorescence emission of SYTO^®^ 9 and Propidium Iodide using 488 nm and 569 nm (Ar-Kr laser) as the excitation source, respectively. The biofilm on sealer disc were divided into four sections using a black permanent marker on the bottom surface of the coverglass-bottom dish (SPL LifeSciences, Gyeonggi-do, Korea). Biofilm structure was examined in three different areas in each section of the biofilm. Biofilm inhibition was expressed by mean [standard error(se)] values of total biovolumes (μm^3^/μm^2^) and mean percentages of red (dead), green (live) and orange (unknown) biovolumes. The images were reconstructed using ImageJ software and analysed using bioImage_L [25].

### 2.3. Determination of pH

Of each freshly prepared sealer (*n* = 12, 3 per group with the experiment performed in triplicates), 200 μL was placed at the base of individual wells of a sterile 12-well flat bottom plate (Star Lab, Milton Keynes, UK) using a 1-mL sterile syringe. 10 mL of HBSS (Gibco®, Grand Island, NY, USA) was immediately added to each well and stored at 37 °C. The HBSS was replaced at each endpoint (15, 30, 60, 120 min and 1, 3, 7, 14, 28 days). The collected HBSS solution was transferred to a glass vial (Fisherbrand, Loughborough, UK) and vortexed for one minute. Determination of the HBSS solution pH was performed at room temperature using a pH meter (FiveEasy Cond Meter F30, Mettler Toledo, Greifensee, Switzerland) and a temperature compensated pH electrode (pH electrode InLab Easy, Mettler Toledo, Greifensee, Switzerland) previously calibrated at five points (pH 2.00, pH 4.01, pH 7.00, pH 9.21, pH 12.00) using buffer solutions (Technical Buffer Solutions, Mettler Toledo, Greifensee, Switzerland). The mean and standard deviation of each were calculated.

### 2.4. Statistical Analysis

A normality test (Kolmogorov–Smirnov) was performed for the examination of data distribution. Analysis of variation (ANOVA) with post-hoc Tukey test was used for data analysis and comparison of the quantifiable viable counts amongst all groups of the DCT and multispecies biofilm inhibition test. The Kruskal–Wallis (nonparametric) test was performed for data analysis of total biovolumes (μm^3^/μm^2^). The overall analysis was performed with SPSS software (version 26, IBM, SPSS Inc., Chicago, IL, USA). In all tests, the level of statistical significance was set at *p* < 0.05.

## 3. Results

### 3.1. Direct Contact Test

**Figure 1:**
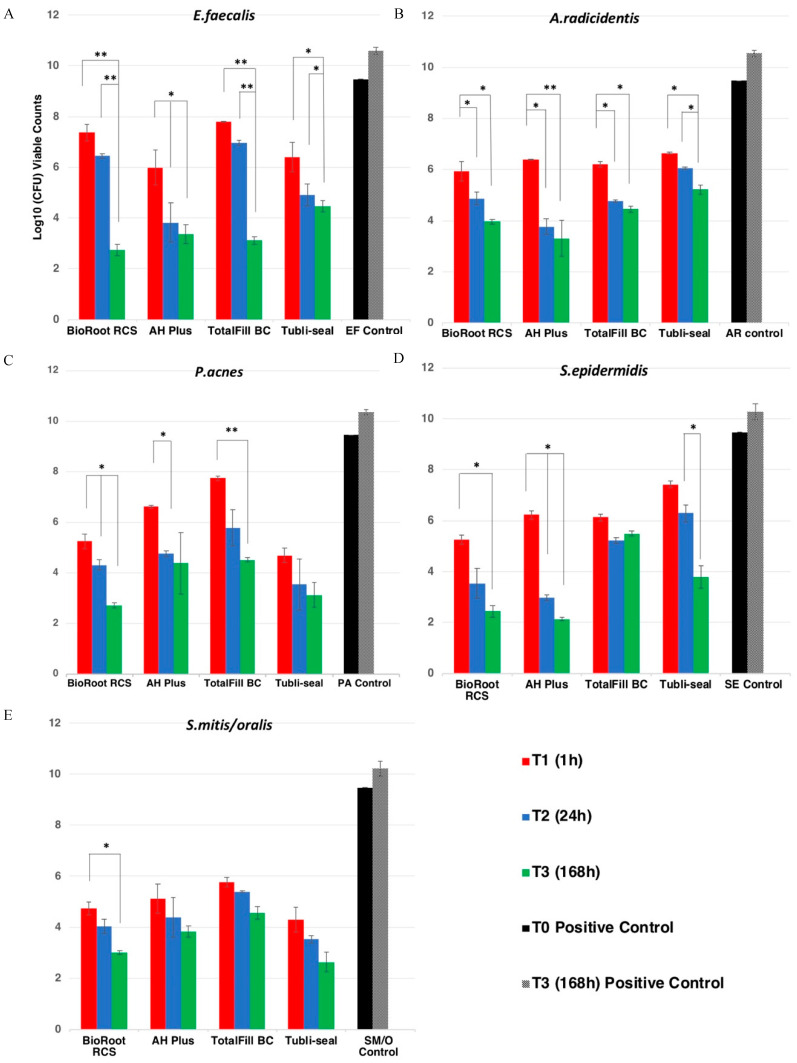
Quantitative viable counts of (**A**) *E. faecalis* (EF), (**B**) *A. radicidentis* (AR), (**C**) *P. acnes* (PA), (**D**) *S. epidermidis* (SE) and (**E**) *S. mitis/oralis* (Sm/o) following direct contact, comparing the effectiveness of each sealer groups at 1 h (T1), 24 h (T2) and 168 h (T3). Black columns represent the control for each bacterium, * (*p* < 0.05), ** (*p* < 0.01). The data are expressed as the mean number of bacteria (±standard error) as log10 (CFU per sample mL^−1^). Negative control group presented nondetectable viable counts at all time points.

The results of the DCT are shown in Figure 1 and Table 3. All negative control group specimens presented nondetectable viable counts at all time points. The bacterial concentration of the positive control group (*E. faecalis*, *A. radicidentis*, *P. acnes*, *S. epidermidis* and *S. mitis/oralis*) at the start of DCT was 3 × 10^8^ mL (9.47 log_10_ CFU mL^−1^). After seven days of growth, the log_10_ CFU values were increased *E. faecalis* (10.59 ± 0.14); *A. radicidentis* (10.55 ± 0.12); *P. acnes* (10.37 ± 0.09); *S. epidermidis* (10.28 ± 0.31) and *S. mitis/oralis* (10.22 ± 0.29).

#### 3.1.1. Effect of Sealers on *Enterococcus faecalis*

At T1 (1 h), AH plus (5.98 ± 0.68) exhibited a significant reduction of detectable viable counts when compared to TotalFill BC (7.71 ± 0.02) and BioRoot RCS (7.36 ± 0.32) (*p* < 0.05). At T2 (24 h), all sealer groups showed a reduction of viable counts, but there were no significant differences between the sealer groups (*p* > 0.05). At T3 (168 h), BioRoot RCS (2.74 ± 0.21) presented the lowest value of detectable viable counts and was significantly more effective in microbial killing than Tubli-seal (4.47 ± 0.23) (*p* < 0.05). However, there was no significant difference when compared to TotalFill BC (3.12 ± 0.15) and AH Plus (3.36 ± 0.36) (*p* > 0.05).

#### 3.1.2. Effect of Sealers on *Actinomyces radicidentis*

All sealers presented a significant reduction of detectable viable counts of *A. radicidentis* at T1 (*p* < 0.05), but no significant differences were found among the groups (*p* > 0.05). At T2, AH Plus (3.77 ± 0.32), BioRoot RCS (4.21 ± 0.24) and TotalFill (4.77 ± 0.05) showed significant microbial killing compared to Tubli-seal (6.06 ± 0.04). All sealers presented lower detectable viable counts at T3 following the order AH plus (3.31 ± 0.71) > BioRoot RCS (3.96 ± 0.09) > TotalFill BC (4.54 ± 0.12) > Tubli-seal (5.22 ± 0.19) when compared to the positive control group (*p* < 0.05), but no significant differences were present between the sealer groups (*p* > 0.05).

#### 3.1.3. Effect of Sealers on *Propionibacterium acnes*

At T1, all sealers presented a reduction of detectable viable counts of *P. acnes* (*p* < 0.05). There was no significant difference between BioRoot RCS (5.26 ± 0.28) and Tubli-seal (4.69 ± 0.29) (*p* > 0.05), but both sealer groups exhibited enhanced microbial action compared to TotalFill BC (7.75 ± 0.09) and AH Plus (6.63 ± 0.06), with the latter being more effective than the former (*p* < 0.05). At T2, the detectable viable counts for Tubli-seal were the lowest (3.56 ± 1.01), but with no significant difference against the rest of the sealer groups (*p* > 0.05). At T3, BioRoot RCS (2.72 ± 0.11) and Tubli-seal (3.14 ± 0.49) presented similar reduction in viable counts (*p* > 0.05), and no difference was present when Tubli-seal was compared with AH Plus (4.40 ± 1.21) and TotalFill BC (4.53 ± 0.09). BioRoot was more effective than TotalFill BC (*p* < 0.05).

#### 3.1.4. Effect of Sealers on *Staphylococcus epidermidis*

At T1, BioRoot RCS (5.27 ± 0.18), TotalFill BC (6.13 ± 0.13) and AH Plus (6.23 ± 0.16) presented significant reduction of detectable viable counts of *S. epidermidis*. Tubli-seal exhibited limited microbial killing efficacy, which was not statistically significant from the positive control group (*p* > 0.05). At T2, AH Plus (2.97 ± 0.12) and BioRoot RCS (3.54 ± 0.60) presented lower detectable viable counts and more enhanced microbial action than Tubli-seal (6.29 ± 0.74) and TotalFill BC (5.22 ± 0.11) (*p* < 0.05). At T3, AH Plus (2.13 ± 0.07), BioRoot RCS (2.44 ± 0.23) and Tubli-seal (3.79 ± 0.43) presented significantly lower detectable viable counts than TotalFill BC (5.50 ± 0.10) (*p* < 0.05).

#### 3.1.5. Effect of Sealers on *Streptococcus mitis/oralis*

All sealers presented a significant reduction at T1 when compared to the *S. mitis/oralis* control group (*p* < 0.05). Tubli-seal (4.30 ± 0.47) presented similar reduction in viable detectable counts compared with BioRoot RCS (4.73 ± 0.25), but enhanced microbial killing efficacy compared to TotalFill BC (5.77 ± 0.18) and AH Plus (5.11 ± 0.57) (*p* < 0.05). BioRoot RCS was more effective than TotalFill BC (*p* < 0.05). At T2, Tubli-seal (3.53 ± 0.14) and BioRoot RCS (4.03 ± 0.28) exhibited enhanced microbial killing compared to TotalFill BC (5.39 ± 0.03) (*p* < 0.05), but no significance difference when compared to AH Plus (4.39 ± 0.77). At T3, BioRoot RCS (3.01 ± 0.08) and Tubli-seal (3.34 ± 0.05) presented enhanced microbial killing compared to AH Plus (3.84 ± 0.22) and TotalFill BC (4.56 ± 0.25) (*p* < 0.05). There was no significant difference between BioRoot RCS and Tubli-seal (*p* > 0.05).

### 3.2. Determination of Quantitative Viable Counts of the Biofilms after 14 Days

**Figure 2:**
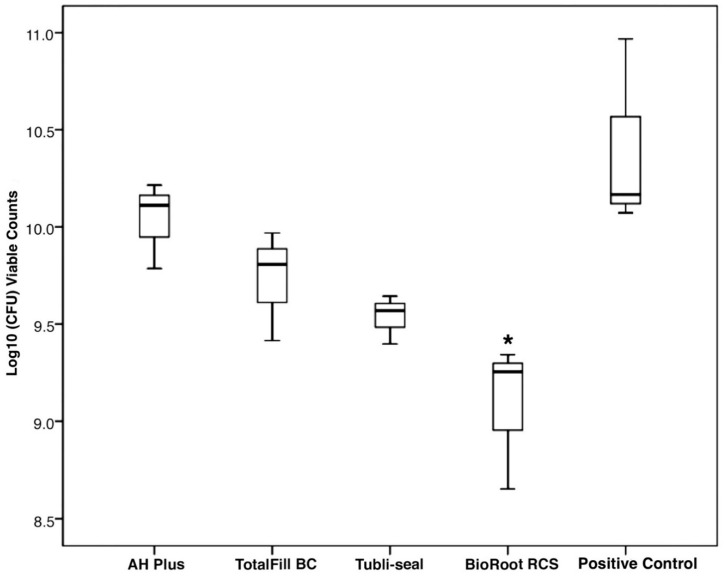
Boxplot showing viable counts (log10CFU) following biofilm growth on set sealer discs, * viable counts significantly less compared to AH Plus and control (*p* < 0.05). Negative control group presented nondetectable viable counts.

The results of the biofilm inhibition test are shown in Figure 2. BioRoot RCS (9.08 ± 0.38) Tubli-seal (9.53 ± 0.13) and TotalFill BC (9.73 ± 0.28) presented lower values, as mean number (±SD) log_10_ CFU, compared to AH Plus (10.04 ± 0.22) and the control (10.4 ± 0.50). BioRoot RCS group presented the lowest log_10_ CFU counts, which was statistically significant compared to AH Plus and the control (*p* < 0.05). No significant difference was observed between BioRoot RCS, Tubli-seal and TotalFill BC (*p* > 0.05). Negative control group specimens presented nondetectable viable counts.

### 3.3. Confocal Laser Scanning Microscopy (CLSM) Analysis of the Effect of Endodontic Sealers on the Inhibition of Multispecies Biofilm

**Figure 3:**
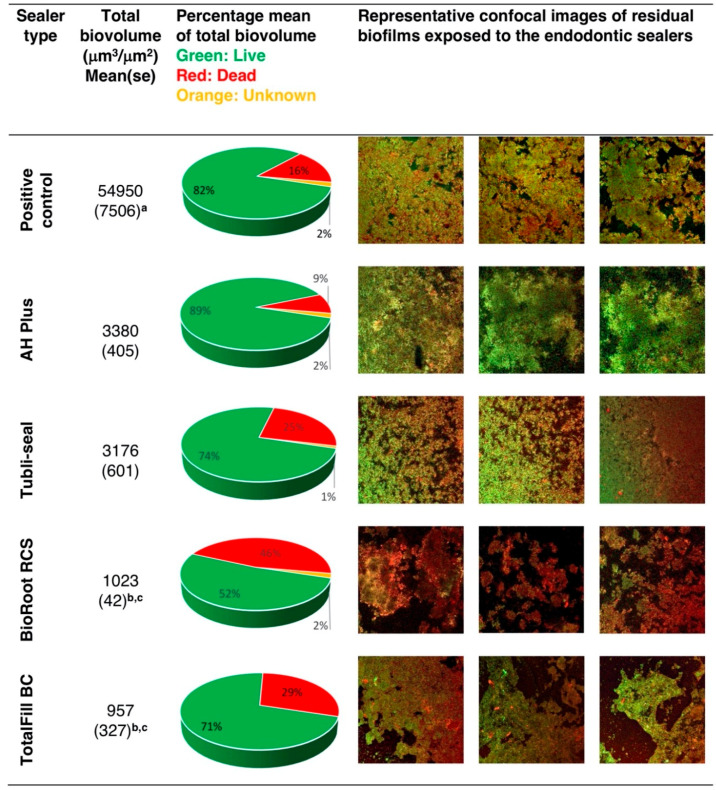
The effects of endodontic sealers on biofilm inhibition. The results represent the remaining mean (±standard error) values of total biovolume (μm^3^/μm^2^) and their respective mean percentages of dead (red), live (green) and unknown (orange) microbial populations. Representative confocal images of residual biofilms are also presented. ^a^ Mean (se) total biovolume values significantly higher compared to all experimental groups (*p* < 0.05) ^b^ Mean (se) total biovolume values of TotalFill BC and BioRoot RCS significantly less compared to AH Plus (*p* < 0.05) ^c^ Mean (se) total biovolume values of TotalFill BC and BioRoot RCS significantly less compared to Tubli-seal (*p* < 0.05).

The effects of endodontic sealers on the inhibition of biofilm growth are presented in Figure 3. All treatment groups presented significantly less total biovolume compared to the positive control group (*p* < 0.05). The lowest mean total biovolume was detectable in TotalFill BC discs, followed by BioRoot RCS. This was significantly less compared to AH Plus and Tubli-seal (*p* < 0.05). BioRoot RCS presented enhanced microbial killing, with the highest mean percentage of 46%, followed by TotalFill BC and Tubliseal with 29% and 25% red (dead) biovolume, respectively. AH Plus showed a mean percentage of 89% green (live) biovolume. Negative control group specimens presented no viable biovolumes.

### 3.4. pH Values of the Endodontic Sealers

**Figure 4:**
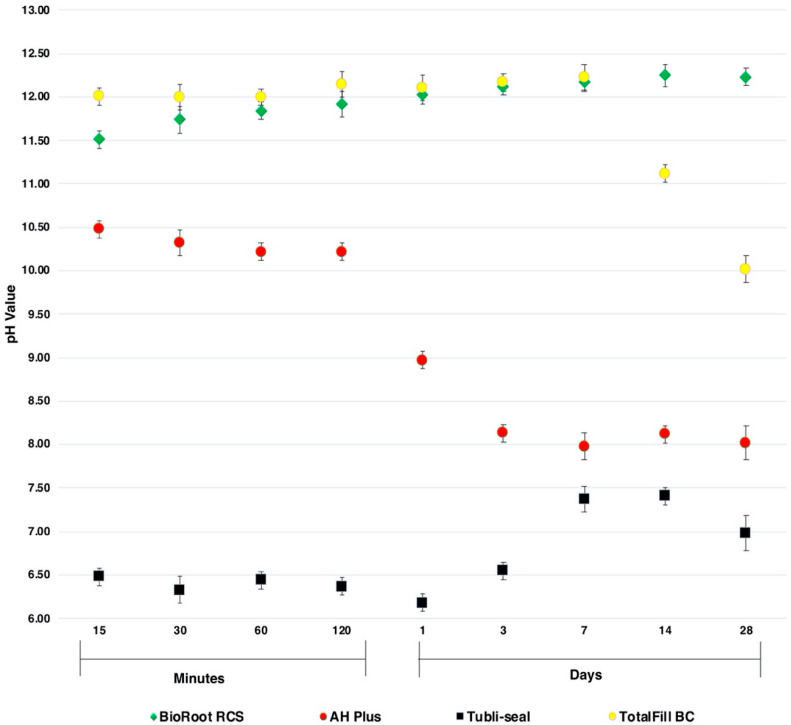
The pH values of the endodontic sealers. The results represented the mean (±standard error) of pH values tested after 15, 30, 60, 120 min and 1, 3, 7, 14, 28 days.

The pH values of the endodontic sealers tested after 15, 30, 60, 120 min and 1, 3, 7, 14, 28 days are shown in Figure 4. TotalFill BC and BioRoot RCS exhibited alkalinisation from the onset. The pH of TotalFill BC remained high for the first 7 days and then decreased over the next 21 days. BioRoot RCS present a gradual rise of pH for the first 7 days and maintained an approximate pH of 12 at day 28. AH Plus induced alkalisation of the HBSS, which was initially higher during the setting phase (pH ~ 10.3), followed by weak alkalizing activity (pH ~ 8) when set. Tubli-seal demonstrated a weak acidification (pH ~ 6.50) of the HBSS for the first three days followed by a neutral pH (pH ~ 7.2).

## 4. Discussion

The antimicrobial efficacy of calcium silicate sealers has been evaluated using the DCT and single species biofilm models [15,16,17,18]. The DCT is a quantitative model which simulates the contact of the sealer in their various phases of setting (freshly mixed or set) and residual bacteria present in the root canal system prior to their ability to organise into a biofilm [14,26]. The single species biofilm model, although accepted, is not wholly representative of the complex polymicrobial biofilm found within infected root canals. Additionally, the endodontic biofilm is subject to nutritional stress leading to increased adherence, alteration of the microorganisms’ cell morphology and surface [27], rendering these microorganisms less susceptible to the effect of antimicrobial agents. The microbes used in this study are prevalent in primary and secondary root canal infections [19,28,29]. To the authors’ best knowledge, this is the first study to investigate the antimicrobial efficacy of freshly mixed endodontic sealers using the DCT against *P. acnes*, *A. radicidentis* and *S. mitis/oralis* and a nutrient-stressed multispecies biofilm model. Considering the relevance of multispecies biofilms in endodontic infections, the advantages of using this model is that it represents the in vivo microbiological environment of the root canal system as closely as possible and permits well-controlled, standardised experimental conditions, enabling complete enumeration of microbial counts. A limitation of the aforementioned model, adapted for this study, was not utilizing substrates of dentine for biofilm growth. However, the use of a dentine model has disadvantages, such as complexities in retrieving the remaining microbial cells adhered on the substrate and deleterious effects on the collagen fibres due to sterilisation of the dentine, which may lead to poorer adhesion of the biofilms.

In the present study, all sealers demonstrated significant antimicrobial activity against the five planktonic bacteria analysed in the DCT. Previous studies evaluating the antimicrobial efficacy of calcium silicate sealers using DCT have reported a pronounced killing effect against planktonic bacteria [11,14,17]. These results are unsurprising due to the fact that these microorganisms are more susceptible to antimicrobial agents in their planktonic cell state [4,30,31].

Substantive long-term antimicrobial effectiveness of calcium silicate sealers demonstrated by the multispecies biofilm test may be positively correlated with calcium ion release [32] and the ability to sustain a high level of alkalizing activity as demonstrated in this study. CLSM analysis further illustrated that BioRoot RCS displayed increased microbial killing compared to TotalFill BC. This could be explained by the ability of BioRoot RCS to sustain longer periods of higher levels of alkalinity, which is in accordance with previous studies [13,33,34]. On the other hand, in the present study, a high initial pH value was obtained by TotalFill BC, which decreased following the sealer’s setting phase. Zamparini et al. [35] demonstrated a similar trend in pH values for Total Fill BC measured over 28 days.

The biological and physiochemical properties of calcium silicate sealers depend on their engineered chemical composition and their setting reactions, which consist of a hydration reaction followed by a precipitation reaction. There are many factors which affect these reactions, such as the inclusion of additives, namely calcium phosphate and calcium chloride, that may alter the properties of calcium silicates [35].

In this study, freshly mixed AH Plus demonstrated high microbial killing but negligible antimicrobial efficacy after setting. The antimicrobial properties of AH Plus might be associated with its alkalinity, with the presence of bisphenol A diglycidyl ether or with the elution of unpolymerized components such as amines released during the setting stage [36,37]. Tubli-seal exhibited antimicrobial properties which may be attributed to its gradual hydrolysis resulting in the release of free eugenol in the medium [38]. In a clinical scenario, this may lead to an irregular porous surface between the core material and canal wall, with a potential for Tubli-seal to resorb and dissolve [39], leading to the possibility of coronal or apical leakage and cytotoxicity [40]. Nonetheless, these materials are regarded as clinically satisfactory, and minimising the sealer interface between the core material and canal wall may curtail these potential drawbacks [41]. The prolonged release of calcium and hydroxyl ions has raised concerns that the calcium silicate sealers exceed the ISO 6876:2012 solubility requirements for endodontic sealers when performed in distilled water [42,43]. Conversely, a recent study found the solubility of calcium silicate sealers was in accordance with the ISO 6876:2012 requirement over a period of six months after storage in PBS [34]. This may be explained by the ability of calcium silicate sealers to form and deposit hydroxyapatite through the release of calcium ions and compensate for any potential drawback of their soluble nature in the long term [13]. Successful endodontic treatment outcomes are dependent on multiple factors, which are strongly influenced by the ability to inhibit microbial colonisation in the root canal. Endodontic sealers possessing various ideal characteristics, such as antimicrobial activity, biocompatibility, sealing ability and handling properties, may positively impact desirable clinical outcomes. The single cone technique, incorporating the use of calcium silicate sealers, has demonstrated promising clinical results [44,45]. Further research, however, is required to investigate calcium silicate sealers, particularly the balance between solubility and their bioactivity through prospective randomised clinical trials.

## 5. Conclusions

Within limitations of this study, freshly prepared endodontic sealers exhibited antimicrobial activity against planktonic bacteria. Calcium silicate sealers showed effective biofilm inhibition capacity and microbial killing, with strong alkalizing activity compared to epoxy-based and zinc oxide-eugenol-based sealers.

## Figures and Tables

**Table 1:** The chemical composition of endodontic sealers tested in this study.

Sealer	Type	Availability	Composition
TotalFill BC	Calcium silicate	Preloaded syringe	35.0−45.0% Zirconium Oxide 20.0−35.0% Tricalcium silicate 7.0−15.0% Dicalcium silicate 1.0−4.0% Calcium hydroxide
BioRoot RCS	Calcium silicate	Hand-mix presentation	(a) Powder: Tricalcium silicate, zirconium oxide, Povidone (b) Liquid: Aqueous solution of calcium chloride and polycarboxylate
AH Plus	Epoxy resin	Mixing syringe	(a) Epoxide paste: Diepoxide, Calcium tungstate, Zirconium Oxide, Aerosil, Pigment (b) Amine Paste:1-adamantane-amine, N,N′-dibenyl-5-oxa-nonandiamine-1-9,TCD-Diamine, Calcium tungstate, Aerosil, Silicone oil
Tubli-seal	Zinc Oxide Eugenol	Mixing syringe	(a) Accelerator: 10−50% eugenol, 20% 5,5′-diisopropyl-2,2′-dimethylbiphenyl-4,4′-diyl dihypoiodite <2.5% zinc acetate dihydrate (b)Base: 50−80% zinc oxide,1−30%, Petroleum, barium sulphate, Starch

**Table 2:** Setting times of the endodontic sealers as stated by the manufacturers.

Sealer	Setting Times
TotalFill BC	4–10 h
BioRoot RCS	4 h
AH Plus	24 h
Tubli-seal	2 h

**Table 3:** Quantitative viable counts of *E. faecalis* (EF), *A. radicidentis* (AR), *P. acnes* (PA), *S. epidermidis* (SE) and *S.mitis/oralis* (Sm/o) following direct contact with each sealer at 1 h (T1), 24 h (T2) and 168 h (T3) comparing the effectiveness between the sealer groups. The values are given as the mean number of bacteria (±standard error) as log10 (CFU per sample mL^−1^).

Bacteria	Sealers	T1, Mean ± SE	T2, Mean ± SE	T3, Mean ± SE
EF	BioRoot RCS	7.36 (0.32)	6.45 (0.09) *	2.74 (0.21) * *****
	AH Plus	5.98 (0.68) * ** ****	3.81 (0.77) *	3.36 (0.36) *
	TotalFill BC	7.71 (0.02)	6.97 (0.1)	3.12 (0.15) *
	Tubli-seal	6.40 (0.58) *	4.91 (0.44) *	4.47 (0.23) *
AR	BioRoot RCS	5.42 (0.38) *	4.21 (0.24) * *****	3.96 (0.09) *
	AH Plus	6.39 (0.02) *	3.77 (0.32) * *****	3.31 (0.71) *
	TotalFill BC	6.21 (0.1) *	4.77 (0.05) * *****	4.54 (0.12) *
	Tubli-seal	6.65 (0.03) *	6.06 (0.04) *	5.22 (0.19) *
PA	BioRoot RCS	5.26 (0.28) * *** ****	4.33 (0.21) *	2.72 (0.11) * ****
	AH Plus	6.63 (0.06) * ****	4.77 (0.12) *	4.40 (1.21) *
	TotalFill BC	7.75 (0.09)	5.80 (0.70) *	4.53 (0.09) *
	Tubli-seal	4.69 (0.29) * *** ****	3.56 (1.01) *	3.14 (0.49) *
SE	BioRoot RCS	5.27 (0.18) * *****	3.54 (0.60) * **** *****	2.44 (0.23) * ****
	AH Plus	6.23 (0.16) * *****	2.97 (0.12) * **** *****	2.13 (0.07) * ****
	TotalFill BC	6.13 (0.13) * *****	5.22 (0.11) *	5.50 (0.10) *
	Tubli-seal	7.42 (0.13)	6.29 (0.32) *	3.79 (0.43) * ****
Sm/o	BioRoot RCS	4.73 (0.25) * ****	4.03 (0.28) * ****	3.01 (0.08) * *** ****
	AH Plus	5.11 (0.57) *	4.39 (0.77) *	3.84 (0.22) *
	TotalFill BC	5.77 (0.18) *	5.39 (0.03) *	4.56 (0.25) *
	Tubli-seal	4.30 (0.47) * *** ****	3.53 (0.14) * ****	3.34 (0.05) * *** ****

* Values significantly less than their respective positive bacteria control group (*p* < 0.05), ** Values significantly less than BioRoot (RCS) (*p* < 0.05), *** Values significantly less than AH Plus (*p* < 0.05), **** Values significantly less than TotalFill BC (*p* < 0.05), ***** Values significantly less than Tubli-seal (*p* < 0.05).
